# TMDlib and TMDplotter: library and plotting tools for transverse-momentum-dependent parton distributions

**DOI:** 10.1140/epjc/s10052-014-3220-9

**Published:** 2014-12-20

**Authors:** F. Hautmann, H. Jung, M. Krämer, P. J. Mulders, E. R. Nocera, T. C. Rogers, A. Signori

**Affiliations:** 1 Rutherford Appleton Laboratory, Oxford, UK; 2 Department of Theoretical Physics, University of Oxford, Oxford, UK; 3 DESY, Hamburg, Germany; 4 University of Antwerp, Antwerp, Belgium; 5 Department of Physics and Astronomy, VU University Amsterdam, Amsterdam, The Netherlands; 6 Nikhef, Amsterdam, The Netherlands; 7 Università degli Studi di Genova, INFN, Genoa, Italy; 8 C.N. Yang Institute for Theoretical Physics, Stony Brook University, Stony Brook, USA; 9 Department of Physics, Southern Methodist University, Dallas, TX 75275 USA

## Abstract

Transverse-momentum-dependent distributions (TMDs) are extensions of collinear parton distributions and are important in high-energy physics from both theoretical and phenomenological points of view. In this manual we introduce the library ${TMDlib }$, a tool to collect transverse-momentum-dependent parton distribution functions (TMD PDFs) and fragmentation functions (TMD FFs) together with an online plotting tool, TMDplotter. We provide a description of the program components and of the different physical frameworks the user can access via the available parameterisations.

## PROGRAM SUMMARY

*Computer for which the program is designed and others on which it is operable:* any with standard C++, tested on Linux and Mac OSX systems

*Programming Language used:* C++

*High-speed storage required:* No

*Separate documentation available:* No

*Keywords:* QCD, TMD factorisation, high-energy factorisation, TMD PDFs, TMD FFs, unintegrated PDFs, small-$x$ physics.

*Other programs used:* LHAPDF (version 6) for access to collinear parton distributions, Boost (required by LHAPDF version 6), Root (version higher than 5.30) for plotting the results

*Download of the program:* http://tmdlib.hepforge.org

*Unusual features of the program:* None

*Contacts:* H. Jung (hannes.jung@desy.de), E. Nocera (emanuele.nocera@edu.unige.it), A. Signori (asignori@nikhef.nl)

*Citation policy:* please cite the current version of the manual and the paper(s) related to the parameterisation(s).

## Introduction

The Quantum Chromodynamics (QCD) interpretation of high-energy particle reactions requires a simultaneous treatment of processes at different energy scales. Factorisation theorems provide the mathematical framework to properly separate the physical regimes. For instance, when two protons collide in a Drell–Yan (DY) event the high-energy partonic cross section is described with a perturbative QCD expansion and the soft physics underlying the structure of the hadrons is treated with parton distribution functions (PDFs), supplemented by QCD evolution. “Evolution”, in this context, refers to the scale dependence of parton distributions (and similar non-perturbative objects) that arises in a detailed treatment of factorisation in QCD perturbation theory. A classic example of a consequence of QCD evolution is the violation of Bjorken-scaling in inclusive deep-inelastic lepton-hadron scattering (DIS), predicted by the Dokshitzer–Gribov–Lipatov–Altarelli–Parisi (DGLAP) evolution equations [1–3].

The same basic picture applies to other (semi-)inclusive processes, like semi-inclusive DIS (SIDIS), and $e^+e^-$ annihilation into hadrons. A PDF describes the likelihood for finding a parton of a particular momentum inside an incoming hadron. In processes with observed hadrons in the final state, fragmentation functions (FFs) enter to describe the transition from a partonic state to an observed final-state hadron.

For sufficiently inclusive processes, only the component of parton momentum collinear to the momentum of its parent hadron is relevant at leading power (*leading twist*) in the hard scale. Factorisation theorems for such processes are traditionally called *collinear* factorisation theorems. In less inclusive processes, however, sensitivity to the partonic motion transverse to the direction of the parent hadron can become important. In such cases, the PDFs and FFs must carry information about transverse parton momentum in addition to the collinear momentum. One must introduce transverse-momentum-dependent (TMD) PDFs and FFs and use them in the context of new factorisation theorems, called TMD factorisation theorems. TMD factorisation has been formulated for a number of semi-inclusive processes including SIDIS, DY and $e^+e^-$ annihilation [4–16]. For particular processes in hadronic collisions, like heavy flavour or heavy boson (including Higgs) production, TMD factorisation has also been formulated in the high-energy (small-$x$) limit [17–20]. In this context, the functions encoding the hadronic structure are more often referred to as *unintegrated* parton distribution functions (uPDFs), see e.g. Refs. [21–29].

The presence of a large variety of TMD factorisation and evolution frameworks complicates efforts to compare different TMD PDFs/FFs and uPDFs parameterisations. In some cases, the differences arise because different formalisms employ similar TMD concepts, but are tailored to specific physical applications. An example is the difference between the Collins–Soper–Sterman (CSS) style of TMD factorisation discussed in Sect. 2.1 compared with the high-energy TMD factorisation style discussed in Sect. 2.2. The former is designed for semi-inclusive processes differential in a particular physical transverse momentum and with a finite and non-zero ratio between the hard scale and the overall energy. The latter (high-energy TMD factorisation) is designed for the limit of a fixed hard scale and very high energies. Moreover, within each category there are also competing subcategories of approaches. For instances, the detailed phenomenological methods that employ a CSS-style of approach in Refs. [30–37] are rather different.

In this paper, we describe a new tool for collecting different fits and parameterisations into a single library, TMDlib , and the online plotter tool, TMDplotter. Provided that the user takes into account all the possible differences between formalisms, collecting parameterisations for both the objects in TMDlib and TMDplotter will also make phenomenological comparisons easier.

The paper is organised as follows: In Sect. 2, we briefly introduce the theoretical framework for both TMD and high-energy factorisation and evolution. In Sect. 3, we present a concise documentation of the TMDlib library and TMDplotter tool, discussing the basic procedure to readily use them.

## Theoretical framework

In this section, we briefly describe two different commonly-used frameworks for factorisation and evolution of parton distributions. Specifically, we discuss TMD and high-energy factorisation theorems and evolution equations.

### TMD factorisation and evolution

When one hard scale enters a high-energy process (like the invariant mass of the exchanged virtual photon in DIS) and the relevant transverse momenta are integrated over, one applies *collinear* factorisation to separate the hard partonic physics from the soft hadronic physics. When sensitivity to intrinsic transverse momentum is important, one must go beyond the collinear framework to factorise perturbative and non-perturbative dynamics. For example, this is the case in processes with observed transverse momenta in the final states, like SIDIS and DY lepton pair production at low transverse momentum. In these cases the low transverse momentum provides greater access to novel QCD dynamics as compared to the collinear case. If the observable transverse momenta are much larger than $\Lambda _\mathrm{QCD}$, then often the cross section may be expressed entirely in collinear factorisation, though supplemented by transverse momentum resummation.

Feynman rules allow for a decomposition of the cross section into a contraction of hadronic and leptonic tensors. Where applicable, factorisation theorems separate non-perturbative and hard contributions within the hadronic tensor. In the TMD case, distribution and fragmentation functions are introduced, whose properties depend on the polarisations of the target and/or produced hadrons, the partonic polarisations, and the twist order. For example, in fully unpolarised SIDIS at leading twist the hadronic tensor is factorised into a convolution of one unpolarised TMD PDF (for the incoming target hadron) and one unpolarised TMD FF (for the final state hadron):

1 $$\begin{aligned} W^{\mu \nu } \!&\sim \! \mathcal{H}^{\mu \nu }(Q;\mu ) \sum _a \int d^2 \mathbf{b}_{\perp } e^{-i \mathbf{q}_{\perp } \cdot \mathbf{b}_{\perp }} f^{a,T} (x,\mathbf{b}_{\perp };\zeta _f,\mu )\ \nonumber \\&\quad {} \times D^{a \rightarrow h} (z,\mathbf{b}_{\perp };\zeta _D,\mu )\ + Y_\mathrm{SIDIS}(\mathbf{q}_\perp ,Q) \nonumber \\&\quad {} + \mathcal{O}((\Lambda _\mathrm{QCD}/Q)^{p}), \end{aligned}$$

where $\mathcal{H}$ is the hard part, $a$ is the flavour of the struck parton, $T$ is the target hadron, $h$ is the detected hadron, $x$ and $z$ are the light-cone momentum fractions, and $\mathbf{b}_{\perp }$ is the Fourier conjugate of the transverse momentum $\mathbf{q}_{\perp }$. The function $f^{a,T} (x,\mathbf{b}_{\perp };\zeta _f,\mu )$ is a TMD PDF while $D^{a \rightarrow h} (z,\mathbf{b}_{\perp };\zeta _D,\mu )$ is a TMD FF. The scale $\mu$ is a renormalization group scale, $\zeta _{f,D}$ are rapidity evolution scales. $Q$ is the hard scale that enters into the hard vertex. In SIDIS $Q = \sqrt{-q^2}$, where $q$ is the four-momentum of the exchanged virtual photon.

The term $Y_\mathrm{SIDIS}(\mathbf{q}_\perp ,Q)$ is a correction for the region of $q_\perp \sim Q$ where a separation into TMDs is not valid, and all transverse momentum is generated inside the hard scattering. This so-called $Y$-*term* is calculable in collinear factorisation. With it included, the corrections are suppressed by powers of $\Lambda _\mathrm{QCD}/Q$, point-by-point in $\mathbf{q}_\perp$, as indicated by the last term, where $p > 0$. Taking into account all the possible combinations of polarisation (parton, target and detected hadron), there are eight TMD PDFs and eight TMD FFs at leading-twist, although the number of operator combinations could be larger [38, 39]. The expression of the hadronic tensor is modified accordingly [40–42].

TMD parton distributions or fragmentation functions depend on two types of auxiliary scales, $\zeta _{f,D}$ and $\mu$, and they satisfy evolution equations with respect to both of them. The evolution with respect to $\zeta _{f}$ and $\zeta _D$ corresponds to Collins–Soper (CS) evolution and is determined by a process-independent soft factor [9, 15, 31, 43–49]. The scales $\zeta _f,\ \zeta _D$ must satisfy the constraint $\zeta _f \zeta _D = Q^4$. The evolution in $\mu$, instead, is determined by standard renormalisation group methods.

When the energy range covered by the experimental data is not large (see, e.g., Ref. [50, 51]) fits of TMD PDFs and FFs can be performed without taking into account effects induced by evolution. These fits rely essentialy on a simple parton model approach and are oriented towards investigations of hadron structure at a relatively low-energy scale. Recent examples are Refs. [35, 52]. In order to explore the evolution of hadron structure with the energy scale, these fixed scale fits can be incorporated into a Collins–Soper–Sterman (CSS) style of factorisation theorem like Eq. (1), as described in Refs. [53, 54]. There, fixed scale fits from [55–59] are combined with traditional CSS style fits from Refs. [30, 31].

### High-energy factorisation and evolution

A form of TMD factorisation holds at high energy [17, 60, 61] and has been applied to several processes in photon-hadron, lepton-hadron and hadron-hadron collisions. For instance, the high-energy factorisation expresses the heavy-quark leptoproduction cross section in terms of the TMD gluon density via well-prescribed, calculable perturbative coefficients [60]. This framework is extended to deep-inelastic structure functions in Refs. [62, 63]. Perturbative applications of the method include the resummation of small-$x$ logarithmic corrections to DIS to all orders in $\alpha _s$ at leading and next-to-leading $\ln x$ level [62–65]. In hadron-hadron scattering, high-energy factorisation has been applied to processes such as heavy flavour and Higgs boson production [20, 60].

In the framework of high-energy factorisation [17, 60, 61] the DIS cross section can be written as a convolution in both longitudinal and transverse momenta of the unintegrated parton density function $\mathcal{A}\left( x,k_t,\mu \right)$ with off-shell partonic matrix elements

2 $$\begin{aligned} \sigma _j ( x, Q^2 ) = \int _x^1 d z \int d^2k_t \ \hat{\sigma }_j( x, Q^2, { z}, k_t ) \ \mathcal{A}\left( { z},k_t, \mu \right) , \nonumber \\ \end{aligned}$$

where the DIS cross sections $\sigma _j$, ($j= 2, L$) are related to the structure functions $F_2$ and $F_L$ by $\sigma _j = 4 \pi ^2 F_j / Q^2$, and the hard-scattering kernels ${\hat{\sigma }}_j$ of Eq. (2) are $k_t$-dependent.

The factorisation formula, Eq. (2), allows for resummation of logarithmically enhanced $x\rightarrow 0$ contributions to all orders in perturbation theory, both in the hard-scattering coefficients and in the parton evolution, taking into account the full dependence on the factorisation scale $\mu$ and on the factorisation scheme [62, 63].

Realistic applications of this approach at collider energies require matching of $x \rightarrow 0$ contributions with finite-$x$ contributions. To this end, the evolution of the gluon uPDF $\mathcal{A}$ is obtained by combining the resummation of small-$x$ logarithmic contributions [66–68] with medium- and large-$x$ contributions to parton splitting [1–3], according to the CCFM evolution equations [73].

The cross section $\sigma _j$ ($j= 2, L$) is usually computed in a Fixed Flavour Number (FFN) scheme, where the photon-gluon fusion process ($\gamma ^* g^* \rightarrow q \bar{q}$) is included. The masses of the quarks are explicitly included with the light and heavy quark masses being free parameters. In addition to $\gamma ^* g^* \rightarrow q\bar{q}$, the contribution from valence quarks is included via $\gamma ^* q \rightarrow q$ by using CCFM evolution of valence quarks [73–75]. A fit of CCFM uPDFs to the combined DIS precision data [76, 77] has been recently presented in Ref. [75] using the evolution given in Ref. [78]. Earlier CCFM fits to DIS were presented in Ref. [79]. In Ref. [80] the unintegrated gluon distribution has been obtained by means of a saturation ansatz.

## TMDlib documentation

TMDlib is a C++ library which provides a framework and an interface to a collection of different uPDF/TMD parameterisations. The parameterisations of TMDs in TMDlib are explicitly authorised for distribution in TMDlib by the authors. No explicit QCD evolution code is included: the parameterizations are as given in the corresponding references. In the present version of TMDlib no attempt is made to unify grid files and the interpolation procedure; both are those provided by the authors.

The source code of TMDlib is available from http://tmdlib.hepforge.org/  and can be installed using the *standard* autotools sequence configure, make, make install, with options to specify the installation path and the location of the LHAPDF PDF library [81, 82] and the ROOT data analysis framework library [83, 84] (which is used optionally for plotting). If ROOT is not found via root-config, the plotting option is disabled. After installation, TMDlib-config gives access to necessary environment variables.

The up-to-date list of all the available functions can be found at http://tmdlib.hepforge.org/namespaceTMDlib.html, and is also summarized in Tables 2, 3 and 4. The TMDlib calling sequence is: *Initialisation* (selecting the desired uPDFs/TMDs), see Table 2; *Call* (producing the uPDF/TMD for partons at $x$, $\mu$ and $k_\perp$), see Table 3; *Information* (displaying details about the initialised uPDFs/TMDs), see Table 4. Note that function overloading is used to create different methods for the functions devoted to uPDF/TMD initialisation (TMDinit) and call (TMDpdf).

- *Initialisation.* The first step consists in initialising the desired uPDF/TMD set. Initialisation assigns the chosen uPDF/TMD set, specified by its name, an identifying number proper to that set.^1^ This number is stored into memory and called each time the identification of the uPDF/TMD set is needed by any TMDlib internal function. The complete list of uPDF/TMD sets available in is given in Table 1 with the corresponding name, identifier, kinematic coverage, and reference. This list will be constantly updated at http://tmdlib.hepforge.org/pdfsets.html as soon as new uPDF/TMD sets will become available. The TMD fit of Ref. [52] is provided as a Monte Carlo ensemble of $N_{\mathrm {rep}}=200$ equally probable replicas, as both a grid with polynomial interpolation and the analytic form with the *best-fit* parameters for each replica. The user should specify the replica to be initialised and whether he would like to use the grid or the parameterisation via the input variables irep and imode respectively. Through imode it is also possible to select the Fourier transform of the TMD PDF, namely the distribution in transverse coordinate space ($b_T$-distribution). For other uPDF/TMD sets, these options are not available and, if specified, they will be ignored.
- *Call to the distribution. * The second step consists in calling the desired function. Specifically, the light-cone momentum fractions $x^+$ and $x^-$ (often set $x^-=0$) carried by the parton, the parton transverse momentum $k_t$ (in GeV), the energy scale $\mu$ (in GeV) and the flavour code identifying the target^2^ are the input variables. Returned is the momentum weighted parton distribution.

Additional methods, utility routines and examples available in TMDlib are:

- TMDutils: collection of methods used in TMDlib , including functions to get details about the initialised uPDF/TMD set (like $\alpha _s$, $\Lambda _{\mathrm {QCD}}$, number of flavours), see Table 4;
- TMD_test: example program to handle uPDF/TMD distributions;
- TMDplotter: ROOT-based script to plot uPDF/TMD distributions as obtained from TMDlib .

**Fig. 1:**
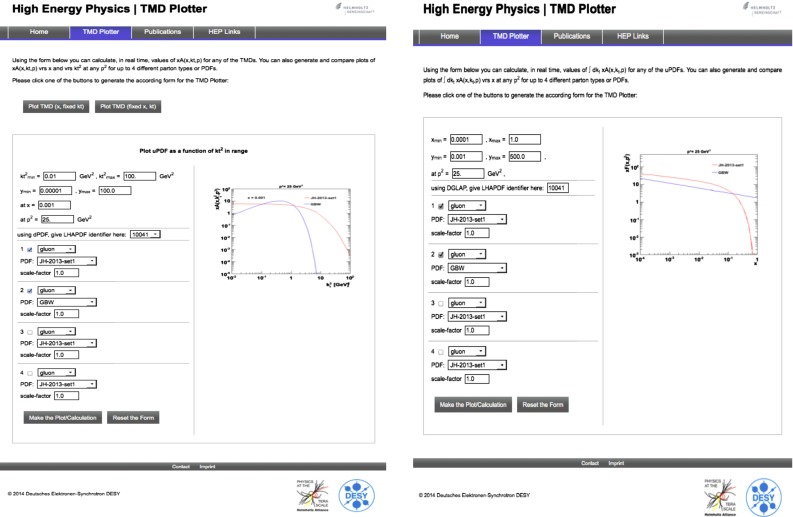
Two snapshots from the online portal TMDplotter for plotting uPDF/TMD distributions: the gluon from the ccfm-JH-2013-set1 set compared to the GBW as a function of $k_t$ (*left*) and $x$ (*right*)

The TMDlib library is released together with the online plotter platform TMDplotter, available at http://tmdplotter.desy.de/. Two snapshots from a typical usage of TMDplotter are shown in Fig. 1: the gluon from the ccfm-JH-2013-set1 set is compared to the GBW as a function of $k_t$ and $x$.

**Table 1:** Available uPDF/TMD parton sets in TMDlib

Parton	uPDF/TMD set	Identifier	$\Lambda ^{(4)}_{qcd}$	$k_t^{cut}$ (GeV)	$Q_0$ (GeV)	Refs.
Gluon	ccfm-JS-2001	101000	0.25	0.25	1.4	[79]
	ccfm-setA0	101010	0.25	1.3	1.3	[79]
	ccfm-setA0+	101011	0.25	1.3	1.3	[79]
	ccfm-setA0$-$	101012	0.25	1.3	1.3	[79]
	ccfm-setA1	101013	0.25	1.3	1.3	[79]
	ccfm-setB0	101020	0.25	0.25	1.3	[79]
	ccfm-setB0+	101021	0.25	0.25	1.3	[79]
	ccfm-setB0$-$	101022	0.25	0.25	1.3	[79]
	ccfm-setB1	101023	0.25	0.25	1.3	[79]
	ccfm-JH-set 1	101001	0.25	1.33	1.33	[85]
	ccfm-JH-set 2	101002	0.25	1.18	1.18	[85]
	ccfm-JH-set 3	101003	0.25	1.35	1.35	[85]
	ccfm-JH-2013-set1	101201	0.2	2.2	2.2	[75]
	ccfm-JH-2013-set2	101301	0.2	2.2	2.2	[75]
	GBWlight	200001	–	–	–	[80]
	GBWcharm	200002	–	–	–	[80]
Quark	ccfm-setA0	–	0.25	1.3	1.3	
	ccfm-JH-2013-set1	–	0.2	2.2	2.2	[75]
	ccfm-JH-2013-set2	–	0.2	2.2	2.2	[75]
	SBRS-2013-TMDPDFs	300001	–	–	1.55	[52]

**Table 2:** The function overload for TMDinit used to initialise uPDF/TMD parton sets

Method	Usage
TMDinit(name)	To initialise the uPDF/TMD set specified by its name name. A complete list of uPDF/TMD sets available in the current version of TMDlib with the corresponding name is provided in Table 1
TMDinit(name,irep)	To initialise a given irep replica in a Monte Carlo uPDF/TMD set specified by its name name
TMDinit(name,irep,imode)	To initialise the uncertainty sets with irep or to initialise a given irep replica in a Monte Carlo uPDF/TMD set specified by its name name and imode:
	$\bullet$ imode=0: the value obtained from the analytic form of the distribution is returned
	$\bullet$ imode=1: the value obtained as a polynomial interpolation on a numerical grid is returned
	$\bullet$ imode=2: the value obtained from the analytic form of the Fourier transform (b-space distribution)

**Table 3:** The function overload for TMDpdf used to call uPDF/TMD parton sets

Method	Usage
TMDpdf(x,xbar,kt,mu, uval,dval,s,c,b,glu)	Void-type function filling the variables uval, dval, s, c, b, glu with the values of $xF(x,\bar{x},k_t,\mu )$ ($F$ is the initialised uPDF/TMD) for valence u-quarks uval, valence d-quarks dval, light sea-quarks s, charm-quarks c, bottom-quarks b, and gluons glu respectively for a proton target. The input variables x and xbar are the light-come momentum fractions $x^+$ and $x^-$ (in some parameterisations the latter is set to zero), kt is the parton transverse momentum $k_t$, and mu is the energy scale $\mu$ (in GeV)
TMDpdf(kf,x,xbar,kt,mu, uval,dval,s,c,b,glu)	As the function above, but for hadron with flavour code kf (kf = 2212 for proton and kf = $-$2212 for antiproton)
TMDpdf(x,xbar,kt,mu)	Vector double-type function returning an array of $13$ variables with the values of $xF(x,{\bar{x}},k_t,\mu )$ ($F$ is the initialised uPDF/TMD): at index $0,\dots ,6$ is $\bar{t},\dots ,\bar{d}$, at index $7$ is the gluon, and at index $8,\dots ,13$ is $d,\dots ,t$ densities for a proton target
TMDpdf(kf,x,xbar,kt,mu)	As the function above, but for hadron with flavour code kf (kf = 2212 for proton and kf = $-$2212 for antiproton)
TMDpdf(x,xbar,kt,mu,xpq)	Void-type function filling an array of $13$ variables, xpq, with the values of $xF(x,{\bar{x}},k_t,\mu )$ ($F$ is the initialised uPDF/TMD): at index $0,\dots ,6$ is $\bar{t},\dots ,\bar{d}$, at index $7$ is the gluon, and at index $8,\dots ,13$ is $d,\dots ,t$ densities for a proton target
TMDpdf(kf,x,xbar,kt,mu, xpq)	As the function above, but for hadron with flavour code kf (kf = 2212 for proton and kf = $-$2212 for antiproton)

**Table 4:** The list of methods included in the TMDutils.cc file

Method	Usage
TMDalphas(mu)	Returns $\alpha _\mathrm {s}$ used in the set initialised by TMDinit(name)
TMDgetLam4( )	Returns the value of $\Lambda _{QCD}$ at $N_f=4$
TMDgetNf( )	Returns the number of flavours, $N_f$, used for the computation of $\Lambda _{QCD}$
TMDgetOrderAlphaS( )	Returns the perturbative order of $\alpha _\mathrm {s}$ used in the evolution of the TMD/uPDF set initialised by TMDinit(name)
TMDgetOrderPDF( )	Returns the perturbative order of the evolution of the TMD/uPDF set initialised by TMDinit(name)
TMDgetXmin()	Returns the minimum value of the momentum fraction $x$ for which the TMD/uPDF set initialised by TMDinit(name) was determined
TMDgetXmax()	Returns the maximum value of the momentum fraction $x$ for which the TMD/uPDF set initialised by TMDinit(name) was determined
TMDgetQ2min()	Returns the minimum value of the energy scale $\mu$ (in GeV) for which the TMD/uPDF set initialised by TMDinit(name) was determined
TMDgetQ2max()	Returns the maximum value of the energy scale $\mu$ (in GeV) for which the TMD/uPDF set initialised by TMDinit(name) was determined
TMDnumberPDF(name)	Returns the identifier associated with the TMD/uPDF set initialised by TMDinit(name)

## Conclusions and feedback

The authors of this manual set up a collaboration to develop and maintain TMDlib and TMDplotter, respectively a C++ library for handling different parameterisations of uPDFs/TMDs and a corresponding online plotting tool. The redistribution of the fits has been agreed with the corresponding authors. The aim is to update these tools with more uPDF/TMD parton sets and new features, as they become available and are developed.

## Footnotes

1 Note that only one set of uPDF/TMD at a time can be called so far.

2 uPDF/TMD parameterisations have been determined for proton or antiproton only so far.
